# Cost-effectiveness of Operative Versus Non-operative Management of Acute Achilles Tendon Ruptures

**DOI:** 10.1007/s11420-019-09684-0

**Published:** 2019-06-08

**Authors:** Jayme C. B. Koltsov, Caitlin Gribbin, Scott J. Ellis, Benedict U. Nwachukwu

**Affiliations:** 1 Department of Orthopaedic Surgery, Stanford University School of Medicine, 450 Broadway Street, Pavilion C, 4th Floor, Mail Code 6342, Redwood City, CA 94063 USA; 2 Department of Orthopaedic Surgery, Hospital for Special Surgery, 535 East 70th Street, New York, NY 10021 USA

**Keywords:** acute Achilles tendon ruptures, cost-effectiveness, cost utility

## Abstract

**Background:**

The management of acute Achilles tendon ruptures is controversial, and most injuries are treated with surgery in the USA. The cost utility of operative versus non-operative treatment of acute Achilles tendon injury is unclear.

**Questions/Purposes:**

The purpose of this study was to compare the cost-effectiveness of operative versus functional non-operative treatment of acute Achilles tendon ruptures.

**Methods:**

A Markov cost-utility analysis was conducted from the societal perspective using a 2-year time horizon. Hospital costs were derived from New York State billing data, and physician and rehabilitation costs were derived from the Medicare physician fee schedule. Indirect costs of missed work were calculated using estimates from the US Bureau of Labor Statistics. Rates of re-rupture, major and minor complications, and the associated costs were obtained from the literature. Effectiveness was expressed in quality-adjusted life years (QALYs). For the base-case analysis, operative and non-operative patients were assumed to have the same utilities (quality of life) following surgery. Deterministic and probabilistic sensitivity analyses were conducted to evaluate the robustness of model assumptions.

**Results:**

In the base-case model, non-operative management of acute Achilles tendon ruptures dominated operative management, resulting in both lower costs and greater QALY gains. The differences in costs and effectiveness were relatively small. The benefit of non-operative treatment was 1.69 QALYs, and the benefit of operative treatment was 1.67 QALYs. Similarly, the total cost of operative and non-operative management was $13,936 versus $13,413, respectively. In sensitivity analyses, surgical costs and days of missed work were important drivers of cost-effectiveness. If hospitalization costs dropped below $2621 (compared with $3145) or the hourly wage rose above $29 (compared with $24), then operative treatment became a cost-effective strategy at the willingness-to-pay threshold of $50,000/QALY. The model results were also highly sensitive to the relative utilities for operative versus non-operative treatment. If non-operative utilities decreased relative to operative utilities by just 2%, then operative management became the dominant treatment strategy.

**Conclusion:**

For acute Achilles tendon ruptures, non-operative treatment provided greater benefits and lower costs than operative management in the base case; however, surgical costs and the economic impact associated with return to work are important determinants of the preferred cost-effective strategy.

**Electronic supplementary material:**

The online version of this article (10.1007/s11420-019-09684-0) contains supplementary material, which is available to authorized users.

## Introduction

Acute Achilles tendon rupture is the most common tendinous injury of the lower extremity, with reported incidence rates between 7 and 40 per 100,000 person-years; the increasing incidence is likely due to greater participation in sports at older ages than in previous generations [10, 11, 15, 17, 26, 36].

The preferred treatment of acute Achilles tendon ruptures in the USA has traditionally been operative management due to its lower reported rates of re-rupture when compared with non-operative treatment [6, 35, 39]. Additionally, operative treatment of Achilles tendon rupture has been demonstrated to provide earlier return to work [35]. Despite the benefits of operative management, there are considerable costs associated with surgery and hospitalization and an increased risk for complications such as wound infection and nerve injury [13, 37, 39]. Also, the introduction and increased utilization of accelerated functional rehabilitation strategies have made non-operative management a more attractive treatment option [4, 41]. Consequently, the choice of operative versus non-operative management remains highly controversial.

Within orthopedic surgery and health care there is increased interest in applying cost-effectiveness research and value-based decision-making to choose between various interventions [21–25, 28, 32]. The purpose of this study was to compare the cost-effectiveness of operative and non-operative management of acute Achilles tendon ruptures, both with early functional rehabilitation, by using a cost-utility model from the societal perspective. By taking into account all of the potential benefits and costs of the two treatment strategies and performing sensitivity analyses of the reported ranges of rates of re-ruptures and complications, quality of life, and direct and indirect costs, we hoped to better inform the choice of treatment. Our hypothesis was that the increased complication rate associated with operative management would be offset by a lower re-rupture rate with surgical approaches, making operative management a cost-effective choice of treatment, particularly in younger, more active patients.

## Materials and Methods

The analyses performed as part of this study were in compliance with the reference case recommendations of the US Panel on Cost-Effectiveness in Health and Medicine [31, 33, 40]. The model structure, assumptions, and health state transitions were reviewed by eight members of the foot and ankle service at our institution, and a consensus-based method was used to finalize the economic model structure. A Markov cost-utility analysis was conducted from the societal perspective to evaluate the cost-effectiveness of operative versus non-operative management of acute Achilles tendon ruptures over a 2-year time horizon. Markov models create mutually exclusive health states through which patients transition over pre-defined analytic cycles. Markov cycles typically last for 1 year, at which point patients stay in the same health state or transition to another health state, depending on cycle probabilities. Markov models attempt to simulate reality, but they may not fully capture all possible variability in treatment strategies. Operative treatment was defined as open Achilles tendon repair with an accelerated functional rehabilitation protocol. Non-operative treatment was defined as non-surgical treatment with an accelerated functional rehabilitation protocol. Accelerated functional rehabilitation differs from traditional rehabilitation protocols for Achilles tendon injury in that patients are progressed to early weight bearing, thereby limiting delays in return to work and activities of daily living.

**Fig. 1:**
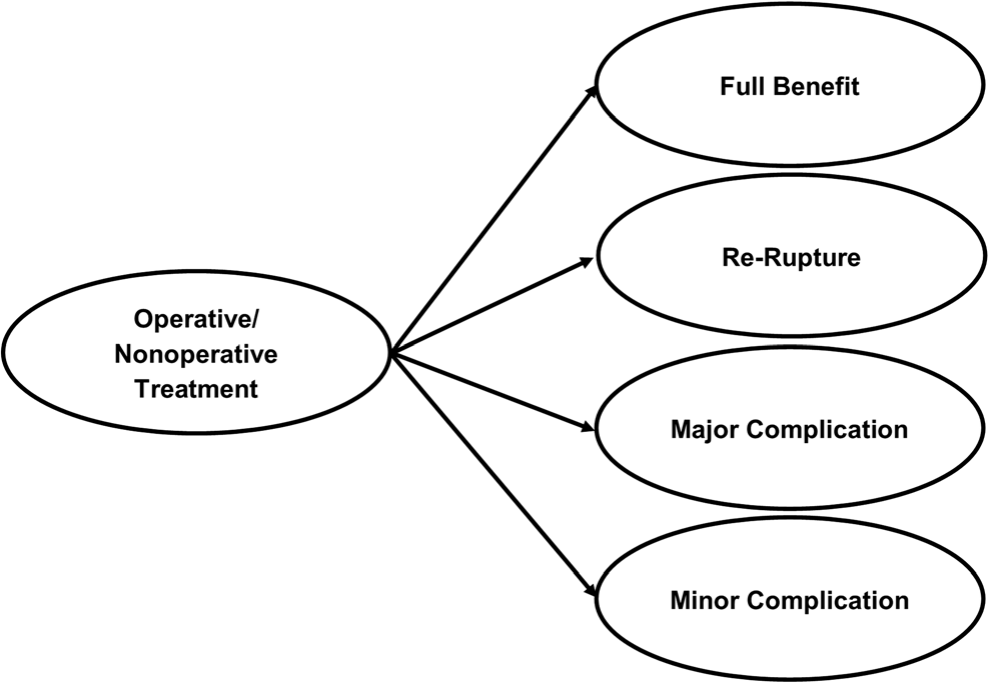
Health states following operative or non-operative treatment for acute Achilles tendon rupture. The probabilities of progression to each of these health states following operative or non-operative management were determined rates reported in meta-analyses of randomized trials.

The model structure contained four health states following treatment (Fig. 1): full-benefit, re-rupture, major complication, and minor complication. The cycle length for the model was 3 months. During the first cycle, the hypothetical patient cohorts undergo operative or non-operative management for their injury, incurring all of the costs associated with these initial treatments. If treatment is successful, patients progress to the full-benefit state. Alternatively, patients move to the re-rupture or complication health states, the probabilities for which were derived from a published meta-analysis [39]. Re-ruptures were assumed to occur within the first 3 months of treatment [5, 12, 18, 41], and all patients experiencing re-rupture were assumed to subsequently undergo surgery, regardless of whether their initial treatment was operative or non-operative. Following surgery for re-rupture, patients could again progress to the full-benefit, re-rupture, major complication, or minor complication health states. The transition probabilities for progressing to these states after re-rupture surgery were assumed to be the same as those for the primary repair surgery.

### Reoperation and Complication Probabilities

Major and minor complications were defined as previously reported in the literature [39]. Major complications included deep venous thrombosis, pulmonary embolism, deep infection, and sural nerve injury; minor complications included superficial infection, transient pain, and painful or hypertrophic scars (Table 1). The probabilities for having a major or minor complication and for re-rupture following each treatment strategy were derived from previous meta-analyses of operative versus non-operative treatment with early weight bearing [34, 39].

**Table 1:** Risk ratios and 95% confidence intervals for operative versus non-operative management with early weight bearing and the calculated rates of re-rupture and major and minor complications [39]

Event	Definition	Relative risk (95% CI)	Operative (%)	Non-operative (%)
Re-rupture		0.40 (0.12, 1.32)	3.8	9.6
Major complication	Deep venous thrombosis Pulmonary embolism Deep infection Sural nerve injury	1.79 (0.64, 5.01)	5.5	3.1
Minor complication	Superficial infection Transient pain Painful/hypertrophic scars	3.54 (0.40, 31.61)	15.4	4.3

### Costs

The initial hospital costs for operative Achilles tendon repair were derived from the New York Statewide Planning and Research Cooperative System (SPARCS) inpatient databases from 2010 to 2014 (Table 2). SPARCS is an all-payer database that contains patient-level information on all inpatient admissions and procedures in New York State. Operative Achilles tendon repairs were identified through the International Classification of Diseases, Ninth Revision, Clinical Modification (ICD-9-CM), via codes 27650 and 27652 for primary procedures and code 27654 for revision procedures, as recommended by the American Foot and Ankle Society. For primary surgery, the total charges were calculated as a weighted average of the two codes based on their incidence, and was dominated by code 27650, which was used for more than 99% of the cases. Charges were converted to costs via hospital-specific cost-to-charge ratios from the Healthcare Cost and Utilization Project (HCUP) [8]. All costs were converted to 2014 US dollars [34]. Due to the short, 2-year time horizon, costs were not discounted.

**Table 2:** Costs of hospitalization and physician, physical therapy, and missed work costs associated with operative, non-operative, and re-rupture (operative) treatment for the base-case model. Hospitalization costs were derived from the New York Statewide Planning and Research Cooperative System (SPARCS), surgeon and physician costs were derived from the Medicare physician fee schedules, and the average costs of missed work were derived using the 2014 average US hourly earnings from the Bureau of Labor Statistics [38]

Component	Operative		Non-operative		Re-rupture	
	Units	Cost	Units	Cost	Units	Cost
Hospitalization	1	$3145	0	$–	1	$3944
Surgeon	1 initial visit 1 surgery + visits w/ in 90 days 1 follow-up visit outside 90 days	$810	1 initial visit 4 follow-up visits	$283	1 initial visit 1 surgery + visits w/ in 90 days 1 follow-up visit outside 90 days	$810
Physical therapy	24 sessions	$821	24 sessions	$821	24 sessions	$821
Missed work	8 weeks	$7834	10.5 weeks	$10,282	8 weeks	$7834
Total		$12,609		$11,386		$13,408

Physician costs for the initial hospitalization and physician visits were derived from the Centers for Medicare and Medicaid Services (CMS) physician fee schedules (Table 2) [2]. As per the CMS payment model, the surgeon fee for the operative Achilles repair included both the fee for the surgery and any additional visits within 90 days. Rehabilitation costs were also derived from the CMS physician fee schedules, and both operative and non-operative treatments were assumed to have 24 physical therapy sessions for the base-case model. The indirect costs for missed work were derived from the 2014 median hourly earnings from the US Bureau of Labor Statistics [38]. Time off work following treatment was 8 weeks for the operative group and 10.5 weeks for the non-operative group in the base case. This was based on the most conservative estimates (i.e., smallest difference between treatment groups) reported in randomized trials of early weight bearing after operative versus non-operative treatment [6, 14, 16, 18, 29].

### Quality of Life

For the base case, we conservatively assumed equivalent quality of life following operative and non-operative treatment. Utilities, which are a number ranging from 0 to 1 with 1 representing perfect health and quality of life, were derived from reported EuroQOL-5D values from the literature [5, 27]. Utility assigned was time dependent. We assigned a utility of 0.9 to full-benefit. In months 0 to 3 following either treatment, patients were assigned a utility of 0.7; for months 3 to 6, a utility of 0.8; and 0.9 thereafter. Minor complications were assumed to reduce utility values by 10%, whereas major complications were assumed to reduce utility values by 20%. For the base case, utilities after re-rupture surgery were assumed to be equivalent to primary surgery. Utilities are accrued through each cycle and summed to provide quality-adjusted life year (QALY) values.

### Sensitivity Analyses

Sensitivity analyses were conducted to evaluate the robustness of the model to uncertainty in the input parameters by varying these over plausible ranges from the literature. Deterministic (one-way) sensitivity analyses were conducted to identify threshold values where results from the base-case model would change. Subsequently, probabilistic sensitivity analyses were used to assess the robustness of the model to simultaneous uncertainty in the input parameters. Beta distributions were used for re-rupture and complication rates data, log-normal distributions were used for relative risk parameters, gamma distributions were used for costs, and normal distributions were used for utility metrics [3]. Input parameters were varied by simultaneously sampling from these distributions in 10,000 Monte Carlo simulations. Monte Carlo simulations provide an opportunity to assess how input parameters affect the distribution of final results.

The model was developed and analyses conducted using TreeAge Pro Suite 2016, R2.1 (TreeAge Software, Williamstown, MA, USA), and other basic calculations were conducted with Excel (Microsoft Corporation, Redmond, WA, USA). The incremental cost-effectiveness ratio (ICER) was our principal comparative measure. In accordance with national guidelines for reporting cost-effectiveness results, strategies that were dominated—less effective and more costly or less effective with a higher cost per QALY gained—were excluded from incremental analysis [7]. ICERs were compared with a cost-effectiveness threshold of $50,000 per QALY [19, 20].

## Results

### Base Case

In the base case for our model, non-operative management of acute Achilles tendon ruptures was both less costly and more effective and thus dominated operative management, resulting in both lower costs and greater QALY benefits (Table 3). The total cost of operative management was $13,936 versus $13,413 for non-operative management. Despite the initial $3145 (95% CI $3045–$3244) in surgical costs, the incremental cost of operative treatment ended up being only $523 over non-operative treatment, due to lower indirect costs for missed work days. The benefit of non-operative treatment was 1.69 QALYs, and the benefit of operative treatment was 1.67 QALYs (Table 2).

**Table 3:** Results from the base-case model comparing non-operative and operative treatment. Non-operative treatment dominated operative treatment, meaning that non-operative treatment was associated with both lower costs and greater benefits in quality-adjusted life years (QALYs)

Treatment	Total costs (2014 US$)	QALYs	Incremental costs (2014 US$)	Incremental QALYs	Incremental cost-effectiveness ratio ($/QALY)
Non-operative	$13,413.04	1.69	–	–	–
Operative	$13,936.38	1.67	$523.34	− 0.02	Dominated

### Deterministic Sensitivity Analyses

We performed one-way sensitivity analyses looking at the influence of direct and indirect costs. For direct costs, we found that if hospitalization costs dropped below $2621 (compared with $3145), then operative treatment became a preferred cost-effective strategy at the willingness-to-pay threshold of $50,000/QALY. Indirect costs were related to worker absenteeism due to treatment, and we found operative treatment became the preferred treatment strategy if non-operative treatment resulted in 3.3 more weeks of missed work (compared with 2.5 more weeks of missed work in the base case) at the median 2014 US wage ($24/h). Similarly, we found that if the hourly wage rose above $29 (compared with $24/h), then operative treatment became a cost-effective strategy.

Model results were highly sensitive to differences in utilities between operative and non-operative treatment. If the utilities (quality of life) for non-operative treatment were just 2% less than that for operative treatment, then operative treatment became the preferred cost-effective option.

We compared the relative importance of individual variables and found that the cost-utility model was most sensitive to the aforementioned relative utilities of operative versus non-operative treatment, costs of hospitalization, and indirect costs due to missed work. Varying the relative rates of re-ruptures and complications over the 95% confidence intervals reported in the literature did not change the choice of treatment strategy.

### Probabilistic Sensitivity Analyses

In probabilistic sensitivity analyses simultaneously varying the input parameters, non-operative management was the cost-effective strategy for 71.7% of the simulations at a $50,000/QALY willingness-to-pay threshold and for 69.1% at a $100,000/QALY threshold.

## Discussion

In the base case, non-operative management of acute Achilles tendon ruptures with an accelerated functional rehabilitation protocol provided greater benefits and lower costs relative to operative management and thus was the dominant treatment strategy for acute Achilles tendon rupture (Fig. 1). Therefore, we reject our initial hypothesis, which was that operative management would be the more beneficial and cost-effective treatment. However, the differences in the overall costs (3.8%) and QALYs (0.02) between treatment strategies were small. The model results were highly sensitive to differences in quality of life after operative versus non-operative treatment and were also sensitive to the variations in the hospitalization cost of operative management and indirect costs from missed time off work. Currently, there is a paucity of evidence regarding the quality of life associated with operative versus non-operative treatment of acute Achilles tendon ruptures, and further investigation is needed to better inform the choice of treatment strategy. Cost-utility analyses are useful for developing population-level approaches, and our sensitivity analyses highlight several scenarios and patient populations for which operative treatment may be favored. Surgery at lower cost centers, such as ambulatory surgery centers, may potentially drive down expenses and favor surgical intervention. Further investigation into streamlined care pathways and the costs and safety of ambulatory Achilles repair is therefore warranted. Additionally, the considerable sensitivity of our cost-utility model to the indirect costs from missed work highlights the importance of shared decision making between physicians and patients, as operative treatment may be particularly preferred for patients in physically demanding occupations where Achilles function is critical for productivity, for patients who are higher wage earners, and for patients desiring the earliest possible return to work.

Our results are limited by the data available from the literature and the New York State inpatient databases. The randomized controlled trials included in the meta-analysis comparing operative and non-operative Achilles repair with early weight bearing were considerably heterogeneous in their treatment, bracing, and rehabilitation protocols [5, 16, 18, 39, 41]. As a result, the reported re-rupture and complications rates varied. Nonetheless, our sensitivity analyses varying the model inputs over the ranges reported in these trials demonstrated that our model was robust to variations in the rates of re-ruptures and complications. The cost inputs from SPARCS were derived from reported charges using cost-to-charge ratios, which are known to contain inaccuracies due to providers not always accurately knowing the costs of care and/or reporting charges for business reasons [30]. Future cost and resource-use studies alongside randomized trials comparing operative and non-operative treatment of acute Achilles tendon ruptures will elucidate the cost side of the equation and the value these treatment strategies provide. The current analysis was limited to a 2-year time horizon, as most data is reported for only the first year or two following treatment. Finally, our analysis has limitations intrinsic to health economic models. We applied robust sensitivity analyses and generalizable assumptions; however, our model may not have captured all the treatment variabilities possible for the operative and non-operative management of Achilles tendon injury.

Although there have been prior economic analyses for Achilles tendon rupture, there has been no prior study comparing the cost utility of operative and non-operative treatments. Our results are in contrast with those published previously from an expected-value decision analysis, which weighed the benefits of operative versus non-operative management from the patient perspective but did not take into account the direct and indirect costs associated with these treatment strategies [14]. Furthermore, the previous analysis drew inputs from the literature prior to 2001 and was dominated by studies not using more recent protocols of early functional bracing and weight bearing.

There is significant controversy within orthopedic surgery regarding operative versus non-operative treatment of acute Achilles tendon ruptures. Earlier studies comparing operative and non-operative treatments supported surgical treatment. Meta-analyses by Bhandari et al. and Khan et al. reported significantly lower rates of re-rupture with operative treatment [1, 13]. However, both meta-analyses were limited by significant heterogeneity of the included studies. Subsequently, rehabilitation protocols for Achilles tendon ruptures have evolved and accelerated functional rehabilitation programs have become a mainstay of therapy. A randomized controlled trial by Willits et al. of an accelerated functional rehabilitation protocol found no significant difference between operative and non-operative treatment for functional outcomes such as strength, range of motion, and calf circumference [41]. Additionally, the authors found no significant difference in re-rupture rate, although they were underpowered to detect this difference. Soroceanu et al. performed an updated meta-analysis incorporating more recent studies with accelerated functional rehabilitation protocols [35]. The authors found that if functional rehabilitation was employed, the re-rupture rate was equal for operative and non-operative treatment. Additionally, there was no difference in calf circumference, strength, or functional outcomes. Surgical patients did, however, return to work 19.2 days sooner. Proponents of operative treatment for Achilles tendon rupture suggest that patients undergoing surgical repair may have sporting and strength benefits. An American Academy of Orthopaedic Surgeons current concepts exhibit reported that for athletes with Achilles tendon rupture managed non-operatively, only 37% achieved return to sport and nearly half had noticeable weakness [37]. Furthermore, Heikkinen et al. found that surgical patients demonstrated 10 to 18% greater strength results at 18 months with significantly larger soleal muscle volume [9]. Our current study adds to the rich debate regarding the optimal treatment strategy for Achilles tendon rupture. From a societal cost and quality of life perspective, non-operative treatment is the preferred strategy. We cannot, however, comment on the role of surgery versus non-operative treatment for specific patient populations seeking to maximize strength or return-to-sport outcome.

Non-operative treatment was the dominant management strategy for acute Achilles tendon ruptures from the societal perspective when early functional bracing and weight bearing protocols were used. However, the direct costs associated with the initial surgery and hospitalization and indirect costs associated with return to work are important determinants of cost-effectiveness of operative versus non-operative management, and the differences in total cost and QALY gains between the two treatment strategies were relatively small. Furthermore, operative treatment is cost-effective if it produces incrementally better function and quality of life relative to non-operative management. Further study of repair quality, level, and timing of return to sports and work, surgical location (hospital or ambulatory surgery center), and quality of life following treatment are warranted and will further identify the patient populations most likely to benefit from operative management.

## Electronic Supplementary Material

ESM 1 (PDF 1224 kb)

ESM 2 (PDF 1224 kb)

ESM 3 (PDF 1224 kb)

ESM 4 (PDF 1224 kb)
